# When the infectious environment meets the AD brain

**DOI:** 10.1186/s13024-022-00559-3

**Published:** 2022-08-19

**Authors:** Tal Ganz, Nina Fainstein, Tamir Ben-Hur

**Affiliations:** 1 Faculty of Medicine, Hebrew University of Jerusalem, Jerusalem, Israel; 2 The Department of Neurology, The Agnes Ginges Center for Human Neurogenetics, Hadassah – Hebrew University Medical Center, Jerusalem, Israel

**Keywords:** Alzheimer’s disease, Infection, Pathogen associated molecular patterns, Lipopolysaccharides, Amyloid-β, Neurodegeneration, Neuroinflammation, Microglia

## Abstract

**Background:**

The Amyloid theory of Alzheimer’s disease (AD) suggests that the deposition of Amyloid β (Aβ) in the brain triggers a chain of events, involving the deposition of phosphorylated Tau and other misfolded proteins, leading to neurodegeneration via neuroinflammation, oxidative stress, and neurovascular factors. The infectious theory linked various infectious agents with the development of AD, raising the possibility that they serve as etiological causes of the disease. Are these theories mutually exclusive, or do they coincide?

**Main body:**

In this review, we will discuss how the two theories converge. We present a model by which (1) the systemic infectious burden accelerates the development of AD brain pathology via bacterial Amyloids and other pathogen-associated molecular patterns (PAMPs), and (2) the developing AD brain pathology increases its susceptibility to the neurotoxicity of infectious agents -derived PAMPs, which drive neurodegeneration via activated microglia.

**Conclusions:**

The reciprocal effects of amyloid deposition and systemic infectious burden may lead to a vicious cycle fueling Alzheimer’s disease pathogenesis.

## Background

The neurodegenerative process in Alzheimer’s disease (AD) is considered the consequence of the deposition of misfolded amyloid-β (Aβ) and hyperphosphorylated tau (p-tau) proteins, with histopathological hallmarks that include Aβ-rich extracellular plaques, p-tau-rich neurofibrillary tangles, microgliosis, astrogliosis, and neuronal loss. Aβ is a peptide consisting of about 40 amino acids, formed by sequential cleavages of amyloid β precursor protein (APP) by β-secretase and γ-secretase. In normal subjects, Aβ is released outside the cell, where it is rapidly degraded or removed. However, in aged subjects or under pathological conditions, the metabolic ability to degrade Aβ is decreased, and Aβ peptides may accumulate [1]. The deposition of Aβ peptides is probably one of the earliest pathological events in AD pathogenesis [2]. However, there are still broad discussions on downstream events, triggered by Aβ deposition, which lead to neurodegeneration. Along with the deposition of misfolded protein in the brain, multiple systemic risk factors have been shown to contribute to disease pathogenesis. Among these are infectious agents, which significantly increase the risk of AD. We suggest here a model by which systemic and bacterial amyloids and other Pathogen-associated molecular patterns (PAMPs) accelerate AD brain pathology. While Aβ induces CNS neuroinflammation which is insufficient in itself to cause neurodegeneration, it results in brain visibility to the systemic milieu and increased vulnerability to microbial PAMPs-induced neurotoxicity, leading to neurodegeneration.

## Main text

### Amyloid deposition induces chronic neuroinflammation: a critical, but insufficient driver of disease

#### Aβ does not directly cause neurodegeneration

The amyloid theory, which has been the mainstream explanation of AD pathogenesis, proposed originally that amyloid plaques and their major constituents, the Aβ fibrils, are the direct cause of progressive neurodegeneration in AD. However, multiple studies have raised important issues that undermine the amyloid theory, including the large temporal gap and the lack of good anatomic correlation between accumulation of amyloid deposits, clinical deterioration, and neuronal loss. Pathological studies were unable to prove a direct correlation or causality between Aβ deposition, clinical dementia, and neuronal loss [3]. In close agreement, transgenic mouse models of AD that carry mutated human genes associated with excessive Aβ deposition and familial AD, are characterized by heavy amyloid deposition, but exhibit no- to only mild- loss of cortical neurons, starting at an advanced age [4]. These suggest that Amyloid pathology may be necessary, but insufficient to cause neurodegeneration. Indeed, different neuronal-injury biomarkers were found to be independent of Aβ [5]. Amyloid imaging studies have shown that Aβ starts to accumulate in the brain approximately two decades prior to clinical dementia and reaches saturated levels several years before the clinical presentation of early dementia [6–8]. This provides a wide gap, during which other pathogenic factors may come into effect and cause neurodegeneration. Further studies found that neurodegeneration in AD was better correlated to local deposition of other misfolded proteins, such as Tau in its highly phosphorylated form, and TDP43 [9, 10], rather than with Aβ. It is thought that Aβ promotes the deposition and dissemination of phosphorylated Tau in the affected brain, leading to neurodegeneration [11–13] via several mechanisms, such as a neuroinflammatory process, oxidative stress, and neurovascular factors. However, we suggest here that in addition to promoting Tau pathology, the deposition of Amyloid causes also brain susceptibility to the neurotoxic effect of external (systemic) insults, and in particular to infectious agents – neurotoxicity.

#### Aβ pathology induces a brain immune response

Multiple studies have shown that Aβ activates the brain’s innate immune system. Pathological Aβ deposits are associated with surrounding (plaque-associated) activated microglia [14, 15]. Monomeric and fibrillar Aβ activates microglia directly via the TLR2 receptor [16]. The Amyloid-burdened brain displays activated glial cells, lymphocytes, and macrophages which release large amounts of inflammatory mediators escalating the inflammatory state and exacerbating other AD pathologies [17].

CNS microglia serve as resident phagocytes that dynamically survey the environment, playing crucial roles in CNS tissue maintenance, injury response, and pathogen defense [18, 19]. Microgliosis, described first by Alois Alzheimer himself, was considered initially the consequence of AD pathology rather than a cause [20]. However, accumulating data have proven that neuroinflammation contributes both to disease initiation and progression. The crucial role of microglia in AD pathogenesis was demonstrated by genome-wide association studies (GWAS) that identified genetic loci which are associated with an increased risk of late-onset AD [21]. These studies have shown that the majority of the loci relate to neuroinflammation and are preferentially or exclusively expressed in microglia. It has become clear that microglia are important players in AD pathogenesis, although it is still highly debated whether the microglial function in AD is beneficial, deleterious, or both. The multiple influences of microglia on AD pathogenesis can be explained by the highly complex nature of these cells, which can polarize into a wide spectrum of phenotypes and activation states, some of them have detrimental effects while others are crucial for disease attenuation and neuroprotection. Among these, several studies have identified the disease-associated microglia (DAM) population, which is increased in both transgenic mouse models of AD and human AD postmortem brains. This sub-population of microglia, which highly express Trem2, is associated with phagocytic activity and Aβ plaque clearance, and is beneficial for AD [22–24]. On the other hand, multiple studies suggest that neurotoxic microglia mediate neuronal death in AD [25–27]. Microglial toxic activation has harmful effects both through a loss of beneficial functions and through a gain of deleterious functions such as the production of pro-inflammatory cytokines and oxidative stress.

Thus, Aβ deposition induces already from early stages the development of an inflammatory CNS environment, manifesting with marked microglial activation. However, the apparent neuroinflammatory process is insufficient to cause neurodegeneration. This raises the possibility that additional factors may drive activated microglia to become fully neurotoxic. Here we discuss the notion that in addition to endogenous CNS misfolded proteins’ -induced neurodegeneration, there are exogenous insults, and in particular infectious agents that promote neurodegeneration in brains that are inflicted with AD pathology.

### CNS visibility and vulnerability to systemic insults in AD

Whether Amyloid or other misfolded proteins drive neurodegeneration, these concepts rely on the traditional thought that Alzheimer’s disease pathogenesis is confined to the nervous system, independent of systemic factors. However, increasing evidence suggests strong bilateral interactions between the brain and the systemic environment that are fundamental to disease pathogenesis.

#### Systemic factors exacerbate AD

First, multiple systemic risk factors such as diabetes mellitus, midlife hypertension, hypercholesterolemia, smoking, and cardiovascular disease, are associated with a significant increase in developing Alzheimer’s dementia and account for up to 50% of the morbidity. Multiple experimental models showed that the increased risk is not merely by co-morbidity, but rather the exacerbation of AD brain pathology by these risk factors. Exposure of transgenic mice models that carry human genes associated with familial AD to systemic risk factors, resulted in the acceleration of the specific AD pathology [28–30]. Moreover, systemic risk factors may affect CNS visibility to the systemic milieu. Specifically, increased plasma glucose levels in diabetes mellitus have been associated with altered blood–brain barrier (BBB) transport functions and oxidative stress in CNS micro-circulation. These changes not only lead to local CNS inflammation but are associated also with upregulation and activation of the receptor for advanced glycation end products (RAGE), which transports Aβ from the blood into the brain across the BBB [31], and therefore may increase Aβ deposition in the brain [32, 33].

Second, different studies have indicated that peripheral immune cells, belonging to either the innate or the adaptive immune system, play an important role in AD pathogenesis. It was shown that circulating myeloid cells mitigate the neuroinflammatory response in AD models and that CNS-infiltrating monocyte-derived macrophages facilitate Aβ plaque removal [34, 35]. Furthermore, systemic regulatory T cells (Tregs) may play a role in disease progression. Some studies suggest that depletion of Tregs accelerated the onset of cognitive decline in mouse AD models, while Tregs administration had neuroprotective effects [36, 37]. Contrarily, others have indicated that pharmacological inhibition of Foxp3 + Tregs is followed by Aβ plaque clearance, mitigation of the neuroinflammation response, and reversal of cognitive decline [38].

#### BBB integrity is breached in AD

The BBB is formed by a tightly sealed monolayer of brain endothelial cells, which keeps neurotoxic plasma-derived components, RBCs, leukocytes, and pathogens out of the CNS [39]. It is widely agreed that cerebrovascular dysfunction and vascular pathology contribute to cognitive decline and neuronal loss in AD [40, 41]. However, a major issue of discussion is whether this vascular dysregulation is an early pathologic event responsible for disease development, or a late by-product of the toxic brain environment. There are multiple indications for BBB disruption very early in the course of human AD, as shown by using various imaging biomarkers of BBB integrity, cerebrovascular reactivity, resting CBF, increased cerebrovascular resistance, and accumulation of brain microbleeds, indicating cerebral amyloid angiopathy [42, 43]. Recent neuroimaging studies in individuals with mild cognitive impairment (MCI) and early AD have shown BBB breakdown in the hippocampus and in several grey and white matter regions [44–46], occurring before brain atrophy or dementia. Moreover, in preclinical AD, changes in vascular biomarkers occur before a detectable increase in standard AD biomarkers, including amyloid deposition, decreased cerebrospinal fluid (CSF) levels of Aβ42 (the most amyloidogenic form of Aβ), and increased CSF levels of tau and phosphorylated tau [47]. In close agreement, BBB integrity is compromised in transgenic AD mice from an early stage, even prior to amyloid deposition [39, 48, 49].

This early BBB breakdown suggests that cerebrovascular changes may be a major driver of the disease pathogenesis, and not just an ‘innocent bystander’ occurring as a result of the dysregulated inflammatory brain environment. The causal role of vascular dysregulation on AD pathogenesis has been suggested as the two-hit vascular hypothesis, where damage to blood vessels is the initial insult, causing BBB dysfunction that eventually leads to neuronal injury and Aβ accumulation [50].

Early BBB breakdown in AD creates CNS visibility to systemic insults, particularly to infectious agents and their products. Blood-borne infections of the CNS in immune-competent subjects with a fully functional BBB are the exception. Indeed, the BBB and CSF barriers prevent the unselective diffusion of vascular and cellular components [51]. In agreement, in healthy mouse models, low and medium doses of endotoxin administered peripherally only minimally entered the brain [3]. However, when the BBB is compromised, various pathogens and pathogens-induced molecules can enter the brain through the bloodstream. It was shown that transgenic AD mice exhibited increased susceptibility to BBB disruption following induction of peripheral inflammatory states [48, 53], providing additional explanation to the observation that AD patients are more vulnerable to the effects of peripheral infection than their age-matched, healthy counterparts [54].

We suggest that early cerebrovascular dysregulation in AD may render the CNS visible to systemic infectious agents, which contribute to disease pathogenesis. Specifically, we will discuss how bacterial Amyloids and microbial PAMPs accelerate AD pathology and cause a direct neurotoxic effect.

### The infectious etiology of AD

Among the various systemic factors fueling AD, accumulating evidence imply an association between infections and AD. Systemic infections are associated with long-lasting cognitive decline in patients with pre-existing AD [55, 56]. This has traditionally been viewed as the human analog of sickness behavior, induced in animal models by inflammatory mediators, including pro-inflammatory cytokines and PAMPs, such as endotoxin, and being pronounced in demented patients due to compromised cognitive reserves. However, mounting evidence infers also an association between systemic and CNS infections to the development of AD [57, 58]. Do infectious agents serve merely as risk factors for AD by unknown mechanisms, or do they cause AD pathology directly? We suggest that both the systemic burden of various PAMPs including bacterial amyloids, as well as neuro-invasion of infectious agents may directly accelerate AD brain pathology.

#### Systemic infections and their products are associated with the development of AD

A well-established infectious-related cause of AD is periodontal disease (periodontitis). Various periodontal pathogens, mainly *Treponema denticola*, and *Porphyromonas gingivalis* have been described as potential contributors to AD pathogenesis. Prospective studies indicate that periodontitis is associated with an increased pro-inflammatory state and cognitive decline in AD, independent of baseline cognitive state [59]. The chronic peripheral periodontal infection may elicit a central inflammatory response by two mechanisms. First, periodontal bacteria cause local production of inflammatory molecules, capable of reaching the CNS via systemic circulation [60] and penetrating the dysfunctional BBB. Second, it has been suggested that stimulation of the trigeminal nerve by periodontal disease in the oral cavity, may be transmitted to induce the production of cytokines in the CNS. These cytokines may have a synergic effect with Aβ on activated microglia, causing an amplified reaction favoring AD progression [61].

The huge mass of microbial organisms in the gut, containing more microorganisms than the entire cell population in the brain [62], makes the brain-gut-microbiota axis another important infectious factor in AD pathogenesis. The seemingly silent gut microbiome may produce important effects on the host body (and its brain) during healthy homeostasis and disease [63]. Studies have shown alterations in the gut microbiome in AD patients, with decreased microbial diversity and distinct composition in comparison to age- and sex-matched individuals. In addition, recent studies in transgenic mouse models of AD have demonstrated that manipulation of gut microbiota can influence cerebral amyloid deposition and attenuate neuroinflammation [64, 65], supporting the notion that the resident microbial flora may affect the pathogenesis of AD brain pathology.

#### Microbial PAMPs contribute to AD pathogenesis

Lipopolysaccharide (LPS) bacterial endotoxins are a major component of the outer membrane of gram-negative bacteria, and an important group of PAMPs. Soluble endotoxin is released when bacteria are destroyed but is also released physiologically as outer membrane vesicles [66]. Therefore, a high load of gram-negative bacteria carrying endotoxins in the microbiome is associated with increased levels of endotoxin in the systemic circulation [67]. When released, endotoxin causes inflammatory activation mainly via activating TLR4 on the cell surface of innate immune cells, including microglia. Animal studies have shown that systemic bacterial endotoxins can induce brain inflammation with accompanying inflammatory-cytokine -induced sickness behavior and cognitive dysfunction [68–70]. Furthermore, endotoxin has been shown to exacerbate brain pathology in animal models, specifically Aβ production and aggregation [71] and Tau hyperphosphorylation [71, 72]. These findings are of clinical relevance, as studies found a threefold increase in mean blood endotoxin levels, a 2–threefold increase in brain endotoxin levels in AD patients, and up to a 26-fold increase in hippocampal tissue [68]. Endotoxin is also found in amyloid plaques [73, 74]. Indeed, people with chronic gingival disease (periodontitis) have elevated blood endotoxin [75], a higher risk of AD [76], and a faster rate of cognitive decline [59, 75, 77].

Another major group of PAMPs is TLR2 agonists, derived from gram-positive bacteria and yeasts. For example, Zymosan is a β-Glucan polysaccharide TLR2 agonist derived from the yeast Saccharomyces cerevisiae, and Lipoteichoic acid (LTA), is a TLR2 agonist that is a major constituent of the bacterial wall in Staphylococcus Aureus and other gram-positive bacteria. Thus, TLR2 agonists are produced by multiple systemic infectious agents affecting patients, including chronic gingivitis [78], skin pathogens [79], and gut microbiome [80]. TLR2 agonists are of particular interest since TLR2 serves as a receptor for Aβ-induced microglial activation [81]. TLR2 mediates Aβ ingestion by microglia and its blockage results in extracellular amyloid accumulation [82]. Neurotoxic activation of microglia by TLR2 agonists may be important in AD pathogenesis and neurodegeneration, as disruption of downstream TLR2 signaling prevented the progression of AD pathology and loss of cortical neurons in AD transgenic mice [83].

#### Invasion of infectious agents to the AD brain

Pathogens invading the brain have been widely studied and suggested also as key causative factors in AD development. Among these are viral pathogens, including Herpes simplex virus (HSV1), Cytomegalovirus (CMV), fungi and bacteria including *Chlamydophila pneumoniae*, *Helicobacter pylori*, *Borrelia burgdorferi* and various periodontal pathogens [84–91].

*Porphyromonas gingivalis* (P. gingivalis) is a keystone pathogen in the development of chronic periodontitis. In transgenic mice overexpressing mutated human amyloid precursor protein, oral infection with *P. gingivalis* impaired cognitive function and increased the deposition of AD-like plaques [92]. Furthermore, *P. gingivalis* LPS has been detected in human AD brains, and *P. gingivalis* DNA was present in the CSF of clinical AD patients [93, 94]. The brain load of Gingipains, major virulence factors of *P. gingivalis*, was significantly higher in AD brains compared to non-demented control brains. Moreover, gingipains were shown to colocalize with intraneuronal Aβ and tau tangles. Oral administration of small-molecule gingipain inhibitors significantly reduced *P. gingivalis* load in mouse brain, decreased the host amyloid response to *P. gingivalis* brain infection, and successfully blocked gingipain-induced neurodegeneration [93].

One of the most studied viral pathogens in the context of AD is HSV1. HSV1 DNA was found to be present in the brain of AD patients at significantly higher levels compared to age-matched healthy individuals [95]. Viral DNA is found within senile plaques [96], Aβ deposition and tau abnormalities typical of AD are observed after infection with HSV1 and are diminished following antiviral treatments [96–98]. In agreement, epidemiological cohort studies showed that HSV1 reactivation, indicated by the presence of both anti-HSV IgM and IgG antibodies, almost doubled the risk for AD in comparison to the presence of anti-HSV IgG alone [99].

Another clue for the importance of HSV1 in AD pathogenesis is indicated by the predilection of the virus to the entorhinal cortex and Temporal lobe, co-localizing with areas presenting early AD pathological changes. This overlap was long described and implies a possible causative role for HSV1 infection in early disease stages [100]. The development of initial AD pathology in the olfactory and entorhinal cortices, and the identification of olfactory dysfunction as one of the earliest clinical symptoms of AD [101, 102], laid the basis for the olfactory hypothesis of AD. This hypothesis suggests that foreign agents are transmitted from the nasal cavity into the brain by the olfactory nerve, as a putative mechanism promoting AD pathogenesis. Studies have suggested that the olfactory system serves as a route of HSV1 entry to the brain [103, 104], and identified HSV1 in the olfactory bulb in post-mortem samples from humans [105]. Furthermore, studies have found *Chlamydophila pneumoniae* present in the olfactory bulb of AD patients, as well as in the entorhinal cortex, hippocampus, and temporal cortex [106]. Another viral agent that was shown to enter the brain through the olfactory nerve and potentially accelerate cognitive deterioration is SARS-Cov-2 (COVID-19 virus) [107].

Although early involvement of pathogens, penetrating the olfactory and entorhinal cortices is widely agreed, it is yet to be determined whether they are the primary cause of AD, initiating amyloid deposition and BBB disruption, or whether prior AD changes starting in these areas enable their penetration.

An additional unique mechanism by which invading pathogens, and other exogenous insults, including environmental pollutants, may contribute to AD pathogenesis is the activation of retrotransposons and silent human endogenous retroviruses (HERVs) [108]. Transposable elements (TE) dysregulation and HERVs activation have been associated to neurodegenerative processes. TE dysregulation may contribute to neuronal death in tauopathies, a significant increase in HERVs transcripts was found in AD [109], and differential expression of several retrotransposons was observed in association with burden of neurofibrillary tangle in human AD brains [110]. It has been suggested that ERV activation may stimulate continuously inflammatory responses, perpetuating the chronic inflammatory environment in AD brains.

#### Infectious agents induce amyloid deposition

How do bacterial and viral agents induce AD pathology? First, the increased systemic and CNS burden of microbial PAMPs may increase neuroinflammation in AD. However, there is increasing evidence suggesting that infectious agents may also directly accelerate Aβ deposition. Gut gram-negative bacteria secrete the amyloid protein Curli, which has marked structural similarity to pathological Aβ [111]. Curli is the major constituent of enteric biofilms, inducing both cell–cell and cell-extracellular matrix attachment [112]. Curli creates potent immunogenic complexes that strongly activate immune cells and induce an antibacterial response [113]. Bacterial amyloids are recognized by innate immune cells as a PAMP, leading to their activation via toll-like receptor 2 (TLR2), and CD14 [114]. While this alone can promote neuroinflammation, it has been suggested that microbial components may also accelerate Aβ deposition in the brain [115]. The inoculation of transgenic 5xFAD mice brains with *Salmonella Typhimurium* bacteria resulted in rapid seeding and accelerated Aβ deposition, in closely anatomic localization with the invading bacteria [116]. Given the structural similarity of Aβ and bacterial Curli, and robust Aβ deposition in response to infection, it has been proposed that Aβ is an anti-microbial peptide, and that pathologic Aβ deposition in the AD brain may be a defensive, anti-bacterial response by the brain’s innate immune system [116]. Brain-derived Aβ entraps and neutralizes invading pathogens, and its oligomerization, a critical step in it becoming pathogenic in AD, may also promote its antimicrobial activities [117].

This association may explain also the robust Aβ deposition in response to HSV-1 infection similar to the brain response to bacterial Curli: Aβ oligomers bind HSV-1 envelope glycoproteins and accelerated β-amyloid deposition was observed in response to herpes virus infection of 5xFAD mice or 3D human neural cell cultures [118].

Aβ is produced in peripheral tissues, including platelets [119], skin fibroblasts, skeletal muscles, and cerebrovascular smooth muscle cells [120–122]. Peripherally produced Aβ is secreted into the blood circulation and is able of crossing the blood–brain barrier [123]. It was shown in a parabiosis model that circulating (human) Aβ invaded and accumulated in the brains of wild-type mice (wt), in the form of cerebral amyloid angiopathy and Aβ plaques. Moreover, these led also to neuroinflammation, tau hyperphosphorylation, and neurodegeneration, comprising the full spectrum of AD pathology [124]. Also, transgenic mice expressing human Aβ only in the liver developed pathological features of neurodegenerative disease [125]. Furthermore, the influx of Aβ from the systemic circulation into the brain was enhanced by *P. gingivalis* infection [126]. Chronic systemic *P. gingivalis* infection-induced Aβ accumulation in inflammatory monocytes/macrophages, and in the brain of middle-aged mice [127]. Importantly, systemic bacterial amyloids can invade the brain, and cross seed with neuronal amyloid [128], suggesting that there is probably no pre-requisite for the entire pathogen to invade the CNS. Thus, both systemic and CNS infections induce an increase in Aβ deposition and AD pathology.

### Molecular mechanism of infection-driven neurodegeneration

#### Microbial PAMPs kill cortical neurons

The deposition of Aβ as a defensive anti-bacterial response that creates a highly inflammatory CNS environment may underlie the close association between various infections and the development of AD. Although most research on the infectious etiology of AD has focused on individual pathogens, a growing body of evidence supports the hypothesis of polymicrobial causality. Consequently, the cumulative exposure to multiple pathogens may cause an “infectious burden” that contributes to the development of the disease. We further suggest that the presence of AD pathology causes CNS hyper-vulnerability to the neurotoxicity of microbial PAMPs, a process that is made possible by the combination of chronic Aβ-induced neuroinflammation and impaired BBB integrity. We have shown that microbial TLR2- and TLR4-agonists kill cortical neurons and that brains inflicted with AD pathology are significantly more vulnerable to their neurotoxicity by two mechanisms. First, in transgenic AD mouse models, the compromised BBB enabled penetration of systemically administered microbial PAMP to the CNS [129]. Consequently, we demonstrated that systemically administered PAMPs induce neurodegeneration in 5xFAD mice, but not in wt mice [129, 130]. These findings may indicate that the increased visibility of AD brains to systemic infectious agents and PAMPs may contribute to their increased vulnerability to neurotoxic effects of systemic PAMPs, resulting in increased death of cortical neurons. Second, we showed that direct delivery of microbial TLR2- and TLR4- agonists cause cortical neuronal death in a dose-dependent manner and that brains inflicted with AD pathology exhibit a marked increase in cortical neuron death, as compared to wt brains [130]. Thus, microbial PAMPs both penetrate and exhibit increased toxicity to the AD brain than to the normal, wt brain.

#### Microglia – a key player in inflammation-induced neurodegeneration

How do microbial PAMPS cause neurodegeneration? Our studies suggest that PAMP-induced neurodegeneration is mediated by brain microglia. First, we and others showed a marked increase in TLR2 + and TLR4 + microglia in human AD and murine AD models [129, 131–134]. Second, we showed that either depletion of microglia by direct Intracerebroventricular (ICV) delivery of Minocycline [129] or modulation of microglial neurotoxic phenotype by direct ICV delivery of a retinoic acid receptor α agonist [130] prevents microbial PAMP-induced neurodegeneration. Finally, we showed that PAMP-induced loss of neurons occurs in the microglia-rich frontal cortex, but not in the microglia-poor CA1 and CA3 regions of the hippocampus [130]. Interestingly, PAMP exposure results in acute death of cortical neurons, rather than inducing a chronic neurodegenerative process [129]. These findings support the notion that recurrent and chronic sub-clinical PAMP exposures may result in a cumulative “infectious burden and cause accelerated neuronal loss. Indeed, we showed that 5xFAD mice housed in a natural environment exhibited accelerated neurodegeneration in comparison to 5xFAD mice housed in a specific-pathogen-free (SPF) facility [130], suggesting that these findings are relevant to the natural infectious milieu. The findings of infectious agents- and microbial PAMPs -induced accelerated neurodegeneration in the 5xFAD mouse model raises the question of its relevance to late-onset AD. Importantly, we studied 7-month-old 5xFAD mice, a time point of heavy amyloid burden and AD pathology, but prior to neurodegeneration. While the 5xFAD model has obvious limitations, such as the lack of deposition of other misfolded proteins, this model, and the choice of mouse age in our experiments, may represent a relatively early stage of late-onset AD, with amyloid accumulation and gliosis. Our findings are compatible with the literature on the early involvement of infections in AD pathogenesis, which may affect the aging population who display Aβ deposition at the pre-clinical phase. Importantly, testing this concept in human patients is possible, for example, by identifying patients who display AD brain pathology at the pre-clinical stage, either by Amyloid-PET imaging [135, 136], or by testing AD-specific biomarkers (eg. Aβ and p-tau) in the blood and CSF [137, 138], and examine prospectively whether a high infectious load is associated with brain atrophy.

## Conclusion

**Fig. 1:**
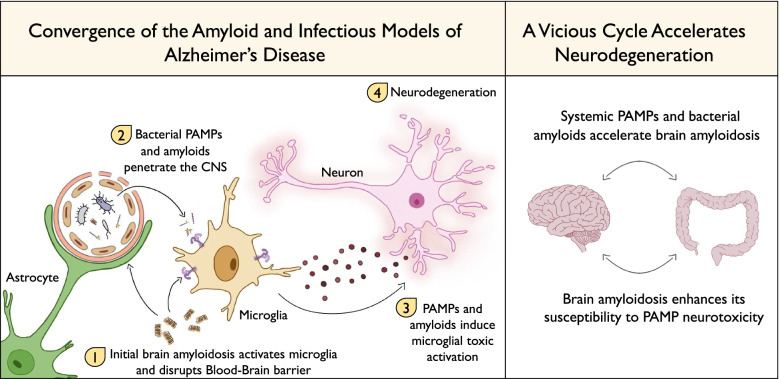
Convergence of the amyloid and infectious theories of Alzheimer's Disease. We suggest that deposition of amyloid β causes increased visibility and susceptibility of the CNS to systemic infectious agents and their components coming from the systemic environment. In turn, infectious agents and PAMPs increase AD pathology and cortical neuron death. These result in a vicious cycle that accelerates neurodegeneration

Within the multitude of systemic drivers and risk factors for AD, and potential interactions between them, we highlight the role of infectious agents and their products in AD pathogenesis, and suggest the convergence of the amyloid and the infectious hypotheses in AD development. We suggest that systemic infectious agents and pathogen-associated molecules can penetrate the AD brain through the leaky BBB (or via olfactory pathways), accelerate Aβ deposition, and act on local microglia, which are already activated and in increased density. These infectious insults further induce neurotoxic activation of microglia, resulting in neurodegeneration. We propose a model (Fig. 1) by which systemic infectious agents induce neurodegeneration, occurring exclusively in vulnerable brain areas with underlying AD pathology. The Amyloid deposition may not cause neurodegeneration by itself, but rather result in brain susceptibility to the neurotoxic effect of infectious agents. This suggests a “hit and run” mechanism, where the infectious agent-derived PAMPs -induced neurodegeneration may be masqueraded, as it occurs in brain areas already displaying marked microgliosis and AD pathology, invisible to the examining pathologist.

While additional studies are necessary to determine which is the initial event in AD pathogenesis, we suggest in our model that viral infections, bacterial amyloids, and other PAMPs, accelerate Aβ deposition, which in turn increases the vulnerability of the brain to their neurotoxic effects. This may create a vicious cycle fueling the disease process. Importantly, the PAMP-induced neurodegeneration is mediated by neurotoxic microglial activation, and is reversible through microglial modulation, thus highlighting their potential role as a therapeutic target.

## Data Availability

Not applicable.

## Abbreviations

AD: Alzheimer’s disease
PAMP: Pathogen associated molecular patterns
Aβ: Amyloid beta
APP: Amyloid beta precursor protein
GWAS: Genome-wide association studies
DAM: Disease-associated microglia
RBC: Red blood cells
MCI: Mild cognitive impairment
CSF: Cerebrospinal fluid
TLR: Toll-like receptor
HSV1: Herpes simplex virus
LPS: Lipopolysaccharide
LTA: Lipoteichoic acid
SPF: Specific pathogen free
WT: Wild type
ICV: Intracerebroventricular
TE: Transposable elements
HERVs: Human endogenous retroviruses
